# Financial autonomy of facilities providing primary care services in low- and middle-income countries: assessing the evidence to inform the development of a typology and conceptual framework

**DOI:** 10.1186/s12913-025-13863-7

**Published:** 2025-12-15

**Authors:** Sophie Witter, Maria Paola Bertone, Lucas Sempé, Quentin Baglione, Hélène Barroy, Justine Hsu, Inke Mathauer

**Affiliations:** 1 Institute for Global Health and Development, Queen Margaret University, Edinburgh, Musselburgh, EH216UU UK; 2 Rebuild Research Consortium, Edinburgh, UK; 3 Agence Européenne pour le Développement et la Santé (AEDES) Brussels, Brussels, Belgium; 4 Health Financing Unit, World Health Organization, Geneva, Switzerland

**Keywords:** Health financing, Financial autonomy, Low- and middle-income countries, Primary care, Purchasing, Public financial management

## Abstract

**Background:**

Provider autonomy is increasingly asserted as an important attribute in health systems, but is rarely interrogated in-depth, particularly at primary care level. This article aimed to examine the current state of evidence on the role of financial autonomy in primary care, focusing on the public sector in low- and middle-income settings (LMICs), and develop a typology and conceptual framework based on it.

**Methods:**

The article draws on mixed methods, including a scoping review of the literature (91 documents), 12 expert interviews and the knowledge of the research team. Findings were also discussed with health financing and public financial management experts at a global meeting in 2023 to deepen the reflections.

**Results:**

In the article, we examine the reforms which have been associated with triggering or at least raising the profile of financial autonomy at primary care level as an important attribute, including strategic purchasing reforms, decentralisation and public financial management (PFM) changes. We highlight important considerations for design and implementation of financial autonomy at primary care level and propose an evidence-based typology structured by the budget cycle, which defines specific dimensions of financial autonomy along a continuum. Finally, we examine what evidence exists on the impacts of financial autonomy and develop a conceptual framework to highlight key considerations in terms of contextual influencers of financial autonomy, prerequisites for it to be deployed, and the potential positive and negative effects of financial autonomy at primary care level. This can be used to encourage future research and inform reform processes in this area.

**Conclusion:**

We conclude that financial autonomy at primary care level can contribute to facility performance, if tailored to contextual factors and supported by accountability mechanisms. However, while financial autonomy is prima facie a positive attribute, the understanding of autonomy over what, for which purposes and by whom is still not clearly addressed in the literature, along with the implications for purchasing and PFM (which is key to enable financial autonomy, as well as being affected by it). This is the first study to our knowledge providing an in-depth understanding of provider financial autonomy at primary care level in LMICs, and moves the field forward with its typology and conceptual framework.

**Supplementary information:**

The online version contains supplementary material available at 10.1186/s12913-025-13863-7.

## Introduction

Provider autonomy involves transferring decision-making rights to facility managers, empowering them to manage various aspects of service delivery [1]. In this article, we specifically focus on financial autonomy, which can be defined as the level of control and influence that health facility managers have to mobilize, allocate, spend and report on financial resources [1]. The topic of financial autonomy of local agencies has long been discussed in the broader new public management literature [2], particularly in service delivery models where local entities were intended to have greater discretion and freedom [1]. Financial autonomy of providers has also been discussed in the health system and health financing literature [3–5]. Recent literature has recognised its importance for strategic purchasing [6–9] and has reflected experiments with mechanisms such as results-based financing [10] and performance-based financing [11], as well as more recent and revitalised interest in financing facilities directly [12–14]. The significance of financial autonomy has also risen in the policy agenda, alongside increasing recognition of the importance of flexible and accountable public financial management (PFM) systems, including in the context of the need for resilient responses to crises [15]

The theory of change for financial autonomy is complex and dependent on various factors. On one hand, increased financial autonomy is believed to promote efficiency and improve outputs (such as quality of care and equity in access) because it empowers providers with responsibility and flexibility over funds, enabling them to procure inputs such as drugs and supplies strategically, align spending with service needs and respond to incentives [12]. On the other hand, this may be nullified if financial autonomy is not supported by flexible budget structures, timely disbursements, agile spending, and accountability mechanisms. Autonomy could also lead to funds misuse, increased corruption or other negative consequences such as profit-maximisation by providers (and hence failure to contain costs and provide rational services) [16] and needs to be balanced with accountability, as too much autonomy without oversight could have unintended consequences.

While financial autonomy has been increasingly identified as important, much of the focus in literature to date has been on hospital autonomy [17] and less on primary care, where different challenges and needs are found and where an explicit theory of change for the role of financial autonomy has not yet been presented. As this is an emerging topic, we undertook a review of literature, deepened by experiential insights, and developed theoretical reflections to move forward the thinking in this area. The objective of this work was to synthesize the existing evidence on granting or expanding/reducing financial autonomy for primary care providers and to inform policy options and guidance, as well as identifying further research needed to fill knowledge gaps.

## Methods

This study was based on a scoping literature review of published and grey literature. Findings were triangulated and enriched with information from consultation with a selection of experts and with the experiential insights of the research team, as detailed below.

### Literature review

#### Search strategy

Given the exploratory approach and the breadth of the topic, the literature search strategy adopted an iterative, purposeful approach to gather all the relevant documentation. The approach used consisted of:

(i) An initial search using the terms “financial autonomy”[All Fields] AND “health”[All Fields] in Google Scholar (screening limited to the first most-relevant 100 hits) and PubMed, where we found 95 hits. After a title and abstract screening, they were reduced to 25;
(ii) Running targeted searches for grey literature in websites including: World Health Organization (WHO), World Bank, Thinkwell, Pan American Health Organization, Organisation for Economic Co-operation and Development, Swiss Development Cooperation, Strategic Purchasing Africa Resource Centre, Results for Development, and Bill and Melinda Gates Foundation;
(iii) Running targeted searches for specific countries identified as particularly relevant in the literature;
(iv) Asking for document suggestions from experts (see consultation process below) and team members;
(v) Taking a snowballing approach by including all relevant references found in selected documents.

#### Inclusion and exclusion criteria

Inclusion criteria included:

- Empirical and normative documents that discuss or reflect on financial autonomy of health care providers, including their context, process and effects
- Documents that focus on public primary care providers in LMICs
- For empirical studies: qualitative, quantitative, and mixed-methods research that use various data collection and analysis techniques, such as surveys, interviews, case studies, statistical models, etc.
- Documents that are written in English, Spanish, Portuguese, French or Italian

Exclusion criteria included the obverse of inclusion, such as:

- Documents focused on autonomy of hospitals and secondary or tertiary care levels
- Documents solely on other (non-financial) autonomy, such as administrative, managerial etc.
- Documents that focus solely on the financial performance or management of health care providers without considering their autonomy
- Documents that examine the autonomy of patients or beneficiaries and of individual health workers (professional autonomy)

### Summary analysis of documents included

**Fig. 1:**
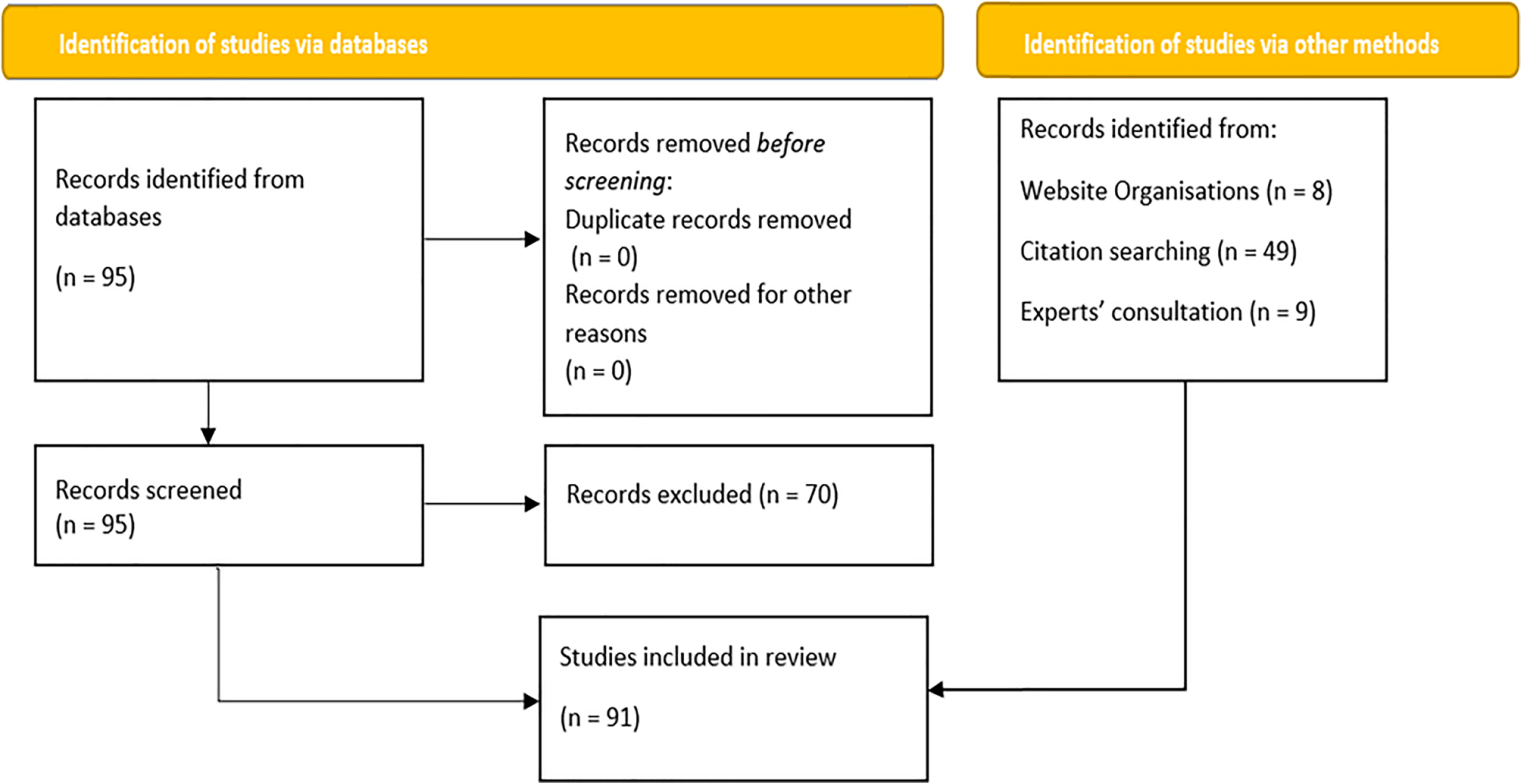
Prisma chart

In the end, we identified and reviewed a total of 91 studies (Fig. 1). The majority of sources focused on sub-Saharan countries (*n* = 51) covering 14 countries, mainly in Tanzania (*n* = 17) and Kenya (*n* = 11). Central Asia was represented by 1 paper in Kazakhstan and 2 in Kyrgyzstan, while Latin America had 3 studies for Peru and 2 papers for Argentina. The bulk of papers were peer-reviewed (*n* = 52) while grey literature represented an important share of sources, being led by reports (*n* = 34).

### Expert consultations

Expert consultation was carried out to complement the literature review, both in terms of experiential insights as well as suggestions for further documentation. The review team also drew on their own professional experiences.

Expert consultation consisted in one-to-one interviews with 12 professionals with a wide geographical expertise (in Eastern Europe, Latin America, Central and South Asia, and Sub-Saharan Africa), identified by the team as experts on the topic. Many are also authors of key documents and have expertise at country level. Although they play multiple roles, the experts can be categorised as senior staff in international organisations (*n* = 2), senior members of think tanks (*n* = 2), national policy advisors (*n* = 3), academics with policy application experience (*n* = 3) and consultants that support strategic purchasing reforms and PFM processes (*n* = 2). All are experts in health financing and PFM and they split evenly between men [6] and women [6].

Experts were contacted via email by the researchers and a mutually convenient time for discussion was set up. Interviews took place in person or remotely via Microsoft Teams. Detailed notes were taken during the interviews (and in some cases, interviews were recorded and automatic transcription generated with the consent of the interviewee).

A topic guide (supplementary file 1) was developed for the expert consultation, based on research questions and the early findings from the literature review. Interviews were semi-structured, allowing focus to vary, depending on the knowledge of the expert. Interviews did not cover any personal, sensitive, or confidential information and were limited to the knowledge that the experts had in their professional role.

### Data extraction and analysis

Relevant information was extracted from each of the selected documents and the consultation notes and compiled into a Word document using a pre-defined structure, based on the research questions and key themes. The information in the document was then iteratively reviewed and refined, by comparing and contrasting information under each theme, and written up following the same structure.

After results were written up, the research team developed a typology for financial autonomy at primary level and conceptual framework, which were shared at the 6^th^ meeting of the WHO Montreux Collaborative on fiscal space, public financial management and health financing in November 2023^1^ and with selected experts for review and feed-back.

### Ethics and consent to participate

As findings are based on literature and on the insights of global experts working in their core area of expertise, no ethical approval was sought for this work. The policy experts are not considered research participants in the conventional sense as they are not the subject of the research, but rather are sharing information on it that they have gained in the course of their practice and employment. Hence, any ethical risks to them are unlikely to arise [18]. Experts gave informed consent to participate. We have anonymised the content of expert interviews.

## Results

In this section, we start by examining reforms which have triggered changes in financial autonomy at primary level, while noting that, despite reforms, a recent study points to the fact that public primary care providers in fewer than 40% of the included LMICs (75 in total) have autonomy to manage and retain income [19]. We then present some of the key elements, challenges and lessons learned in relation to the design and implementation of changes to financial autonomy, followed by synthesising their impact.

### Health sector reforms affecting financial autonomy

#### Strategic purchasing and provider payment reforms

Financial autonomy has been increasingly discussed in recent years in relation to waves of reforms of health financing arrangements, specifically in relation to strategic purchasing [7], and specifically under Performance Based financing (PBF) and Direct Facility Financing (DFF) [20]. These reforms are reported to have triggered many of the changes in autonomy of primary care providers. That is because financial autonomy is inherent in the nature of strategic purchasing which requires facilities to obtain and retain funds and be able to use them with some discretion, in order to achieve better results by addressing challenges with locally-adapted approaches that respond to community health needs (rather than those defined from above) [20, 21]).

There is a vast literature on PBF reforms and their effects, as a systematic review of 2022 reveals [22]. However, this literature often looks at the issue of autonomy in passing. The documents focusing on unpacking the conceptual foundations of PBF stress the role of facility autonomy as essential in order for providers “to be able to respond in a creative way to overcome bottlenecks in attaining results at their level” [21 p. 3] [23]. Similarly, the World Bank’s 2014 PBF Toolkit dedicates a full chapter at the issue of autonomy and states that, “PBF is premised on a substantial degree of health facility autonomy”, which includes financial, but also managerial autonomy and autonomy over human resources and procurement [24]. Evidence from Zambia shows that one of the key drivers of the impact of PBF was it being coupled with increased financial autonomy for facilities (balanced with accountability for both results and use of funds) [10, 11].

DFF reforms are more recent and therefore the literature and empirical evidence are more limited. However, in the 2022 WHO conceptualisation and guidance on DFF [25], facility autonomy is proposed as the first of three key principles. As DFF is promoted as a potentially effective reform, facility autonomy at primary care level becomes an essential lever.

Two issues emerge from literature and experts: (i) the degree of autonomy allowed to primary care facilities varies substantially across projects and reforms [26] and between theory and practice (KII 2); (ii) the increase in autonomy can be short-lived and project-related if it is not fully embedded within the PFM systems or the overall legal frameworks [19, 27, 28] (KII 2). In some countries, governments have recognised the need to put more funds under facilities’ control and allow them more financial autonomy, in particular when also implementing broader health financing reforms which entailed changes in provider payment mechanisms. Most payment reforms have been focused on the hospital sector, but some payment reforms have also targeted primary care, including through capitation and output-based payment approaches. For example, in Burkina Faso, the Free Health Care programme introduced a direct financial flow from the Ministry of Health (MoH) to primary care facilities, allowing facilities to spend funds flexibly to cover operational costs [29]. In Uganda, the grant to facilities for non-wage recurrent costs, coupled with the updating of the PFM frameworks accordingly, also provides for more financial autonomy by facilities. In Nigeria, the Basic Health Care Provision Fund increases availability of funds for primary care providers, although with substantial involvement by state-level agencies. In Indonesia, under certain conditions, primary care centres (*puskesmas*) can become semi-autonomous budgetary units known as *Badan Layanan Umum Daerah* (BLUD), in order to have enhanced control over both allocated and internally generated funds [30].

Yet it is important to note that reforms to purchasing and payment methods do not always trigger changes to primary care providers’ autonomy, and some of the literature points to limited autonomy as an ongoing constraint. For example, in the Philippines, the limited operational and financial autonomy of public primary care providers is seen as preventing them from fully responding to the incentives inherent in the different payment methods of PhilHealth, since health centres and rural health units do not have their own accounting unit to manage income [31].

#### Decentralisation reforms

In decentralised system, such as those in Latin America, the majority of primary health services are managed by subnational institutions (KII 1), which means that the design of intergovernmental conditional transfers is crucial to providing financial autonomy for providers. If well designed and implemented, these can incentivize and enforce sub-national governments to ensure facility autonomy, through fund conditionality and providing technical assistance (especially to less developed regions or provinces).

However, while decentralisation processes may entail changes to purchasing mechanisms and PFM rules, systems, and processes [32], decentralisation does not necessarily enhance the financial autonomy of (primary care) providers and can indeed reduce it [30]. Often new decision-making powers and fiscal controls are extended to subnational levels, but not systematically including providers [33]. For instance, in Kenya decentralisation resulted in a recentralisation of autonomy over financial management from the public health facility level to the local government level [1]. This is because functions previously delegated by central government to providers were shifted to subnational government bodies (counties) [30, 34].

Similarly, in Chile, a capitation-based health budget was introduced in the 1990s. Yet this budget is administered by the municipality, which is responsible for purchasing goods and services, including payment of salaries, required for the operation of the primary care facilities. Therefore, primary care centre directors have only limited autonomy and rural centres are even less autonomous due to their financial constraints [35].

Another typical set of challenges became visible in Peru. As part of its decentralisation reforms in the 1990s, Local Health Administration Communities (CLAS) were established as community-based organisations aimed at giving local oversight and management of primary health facilities, providing full financial autonomy to them, with strong reporting and accountability mechanisms. While CLAS have budgetary authority on paper to allocate funds based on local priorities and healthcare needs, insufficient national resources hamper their financial autonomy, especially in rural and underserved communities where needs are greatest and which CLAS were meant to empower [36, 37].

#### PFM reforms

Recent literature on public financing for health stresses how traditional PFM systems can be a “stumbling block” for facility performance and responsiveness, including limiting facility autonomy [38] and that PFM reforms often focus on a standard set of interventions that are not tailored or responsive enough to the specific needs of the health sector and in particular of primary care facilities [39]. The introduction of financial autonomy at health facilities might be triggered by PFM reforms (outside of the health sector) that focus on improving planning, budgeting, procurement, accounting, and auditing systems (KII 5). However, experts observed that PFM reforms have most often not triggered facility autonomy, because the latter may clash with some basic PFM principles (such as having a single treasury account, the structure of the chart of accounts not recognizing facilities as spending units, and the Integrated Financial Management and Information Systems (FMIS) not being applicable or appropriate to facility-level financial reporting) (KII 9, KII 11). These experts saw the catalyst of change as starting with health financing reforms, which entailed changes to facility autonomy and in turn triggered PFM adjustments. For example, in Burkina Faso, as discussed above, greater flexibility was needed to manage free care payments, which triggered adjustments to the PFM rules and processes for the health sector [29].

The frequent misalignment between the PFM system and health financing arrangements is well known and noted in the normative literature, which highlights how the lack of autonomy for facilities determined by PFM rules is a major constraint to strategic purchasing reforms [39, 40]. Empirical evidence shows that, in some cases, rules that allow facility autonomy, linked to the introduction of a strategic purchasing reform (such as PBF), clash directly with PFM regulations [41]. For instance, in Cameroon, facilities are normally required to return part of their revenues to the central level, although facilities in the PBF scheme should be exempted [42]. In practice, however, managers reported pressures to comply with pre-existing laws. Similarly, in Cote d’Ivoire, changes have been made to strategic purchasing arrangements (i.e., introduction of PBF) without allowing sufficient autonomy for the implementation of those changes beyond the limited PBF/project funds (KII 5) [27]. In Kenya, in order to address the reduced facility autonomy after the decentralisation reform, some counties have developed parallel PFM rules, leading to a patchwork of legal frameworks. In addition, everyday PFM bottlenecks *de facto* constrained autonomy – for example, county health department budget ceilings are not always transparent and not communicated to public health facilities [1].

In addition, rigid line-item budgets do not allow for decisions to reallocate funds according to needs, leading to inefficiencies. Economic classification used by both the Ministry of Health (MoH) and public primary care providers can result in providers being restricted to the same historical input mix, with limited room for optimizing resources and planning investments, and no autonomy to reallocate funds to changing needs during the year. Programme budgeting allows the identification of primary care clearly in the national budget and would allow more flexibility for primary care facilities if ex-ante input-based controls were eliminated. However, the empirical experience points to the fact that, in practice, even when programme budgeting is adopted, it is often applied in addition to the historical economic classification, and/or is used only for budget formulation, but not for the monitoring of budget execution, so that only inputs that were explicitly budgeted for can be procured [28]. According to a recent review on 20 LMICs, its potential has not been fully realised in the health sector due to flaws in reform design and implementation, and limited connection with the requirements for primary care financing [43].

#### Political barriers to increases of financial autonomy for primary care facilities

There is limited empirical evidence in the literature on the political economy dynamics in relation to changes to facility autonomy. However, most experts agreed that financial autonomy is a politically sensitive topic. This is because increased financial autonomy at facility level implies (or might be perceived as) a reduction of influence for the MoH, Ministry of Finance (MoF) and the local government, which would lose control over resource allocation and also over information [38, 44]. The case of Kenya, where changes in autonomy were linked to the decentralisation reforms, show that the newly created local (county) governments were reluctant to relinquish control over facility resources which the new law allowed them, as this represented a major source of revenue at county level [1, 33].

Anecdotal evidence points to the fact that, as a consequence, MoH and central level bureaucrats tend to prefer limited autonomy at facility level in order to maintain power and control over expenditures and activities. As one of the experts pointed out, increasing facility financial autonomy implies a more profound organisational change for the MOHs than for the facilities themselves, because they have to convert their “command and control” leadership model into a more distributed leadership strategy. Specifically, it implies adopting new practices such as the development of a performance management strategy, goals setting and measurement, change management strategies, expanding supervision and managerial training, designing new reporting systems, and last but not least being accountable to the Ministries of Finance for what the facilities decide, for how hundreds of facilities allocate their funds (KII 1).

The MoF might have a more ambiguous position – in some cases, raising technical issues that might question autonomy for facilities, for example in relation to how to pay multiple facilities, or authorisation and control as well as accountability concerns [38] (KII3). However, as many of these technical concerns are increasingly addressed by information and communication technology (ICT) solutions, MoFs may be shifting towards support of such reforms.

At the same time, donors have historically preferred either to deal with the MoH directly (KII3) or to fund through systems that do not allow to support effective autonomy (KII6). However, in some settings, donors have become frustrated with the lack of transparency and accountability of funds beyond the district level, when managed by the DHMT. This is the case in Zambia, where more than 55% of funds spent at the district level cannot be mapped against budgets or spending categories. As a consequence, donors have been supporting reforms to increase direct funding to facilities and facility autonomy, coupled with strong reporting and information systems, in countries such as Tanzania, either through government or parallel systems, where government ones are perceived to be weak [41].

### Design and implementation considerations for financial autonomy

#### Scope and degree of autonomy

There are different functions which can be affected by financial autonomy. It may refer to planning autonomy only, where facilities have a say on how their allocated funds are to be used but do not manage cash directly, which is rather managed on their behalf, for example by district offices. It can expand to autonomy over raising and managing funds, for example, being able to retaining the funds at facility level, rather than sending them to a central treasury, and being able to roll them over from year to year or not. It can cover decisions over expenditure of various kinds, with varying degrees of flexibility in utilising different available resources [45].

Another key design question relates to the exact cost components that are included under financial autonomy, with particular reference to decisions over staff and drug procurement. Despite staff being one of the most important expenses for primary care facilities, decision making over core HRH is usually kept outside the remit of financial autonomy at facility level (including hiring and firing, and decisions on payments) [46, 47]. This is because usually civil servants’ salaries do not go through MoH PFM systems and are frequently managed by a centralised civil service agency or Ministry of Public Service (KII 6, KII 11). On the other hand, autonomy to pay (and therefore hire) contractual staff is found in some settings, for example in the case of PBF funds and user fees that allow facilities to hire local staff (such as cleaners, guards and pharmacy managers) (KII 11). Similarly, concerning drug procurement, while facilities themselves are best placed to order the types and volumes of drugs based on their needs, procuring or purchasing directly by primary care facilities can be inefficient or not possible, and therefore it is often left outside of the scope of financial autonomy for primary care providers (KII 5). However, another expert noted that the two elements of paying for drugs and procuring them could be kept separate. From a PFM perspective, there is no reason why facilities should not pay for their drugs, while from the procurement perspective there is a debate on the advantages and disadvantages of centralised procurement (KII 11).

Country experiences show that primary care facilities tend to face few high-value expenses (such as medical equipment procurement); the remaining is relatively small-value transactions to cover recurrent operational costs (such as consumables, cleaning materials, contracted staff, etc.) (KII 3, KII 6) [45]. Often an 80/20 rule-of-thumb applies, i.e., 80% of operational transactions (such as materials, basic equipment, utilities, repair and maintenance, contract labour) account for 20% of expenses [45]. Given different fiduciary risks, experts pointed to the fact that it might make sense to increase flexibility over those small-value transactions, which represent minimal financial risks but are essential for effectively providing health service.

#### PFM and wider accountability requirements

Much of the literature (for example [12, 48]) assumes that financial autonomy requires primary facilities to be or to become ‘spending units’ (or otherwise called ‘costs centres’), officially recognised within the chart of accounts and reporting as such. Some country experiences have proven that facilities can receive funding without being a spending unit, for example, Burkina Faso [29] and Zambia [49]. This can be a simpler, temporary measure as broader PFM changes are being introduced and retrofitted into legal frameworks (KII 11). Another PFM requirement for facility autonomy is linked to having facility-level bank accounts. Officially, this is a requirement in settings such as Tanzania. But in some settings, it may be feasible to work through ‘cash vouchers’ available at district-level bank accounts, when banks do not exist in some of the facilities locations (for example, in Burkina Faso) (KII 9). A third requirement is to have a robust financial management information system (FMIS) for facility-level reporting. In some countries, the FMIS is supposed to be established down to provider level. However, the design of FMIS does not fit provider-level spending granularity and its implementation does not reach or cover that level of the system (KII 9). Additional systems can be developed to better report and inform on provider-level spending. A key question is the inter-operability of those systems. Recently, Burkina Faso has developed an e-platform for the free care programme, providing detailed reporting of primary care provider spending (KII 9) and connecting it with operational performance.

An essential consideration when tailoring reforms to the context is the availability of supporting infrastructure which is needed to allow autonomy to effectively work for primary care facilities. This includes availability of bank accounts and banking infrastructure (KII 7) as well as ICT infrastructure (electricity, internet connectivity, functioning computer, skills etc.) (KII 4). In some settings, banking infrastructure might not be at all available at primary care level. In Burkina Faso, when facilities where given access to funds with the introduction of the free healthcare reform, those which could not open a bank account were allowed to keep funds as earmarked (*cheque bloqué*) in the bank account of the district health office (KII 11). Some experts have mentioned that mobile money could represent a way forward in the future (KII 11). Similarly, especially in fragile or conflict-affected settings or in extremely rural areas, it is possible that there could be no availability of medicines and other supplies to be purchased autonomously near the facility (KII 7).

The literature emphasizes the importance of designing and implementing effective financial management tools for planning, budgeting, spending and reporting, as well strong oversight structures to support increased financial autonomy [25, 39]. Appropriate accountability systems and expenditure rules emerge as crucial requirements to balance managerial discretion over finances with fiduciary and other risks. Importantly, empowering facilities does not mean leaving them alone and does not eliminate the responsibility of the central level to guide and support facilities. On the contrary, facilities may need more support than before. Autonomy without strategic guidance can bring more fragmentation, inefficiencies and more disconnection between actors (KII 1).

For instance, in Tanzania, internet-based IT solutions such as PlanRep (Planning and Reporting System) and Facility Financial Accounting and Reporting System were introduced to support management functions at facility level as well as oversight by District Health Management Teams (DHMTs) [49]. Evidence from Zambia shows that one of the key drivers of the impact of PBF was it being coupled with increased financial autonomy for facilities, but also balanced with accountability for both results and use of funds [10, 11].

#### Staff and capacity for financial autonomy

A concern shared in the literature and by many experts is about carefully considering staffing needs. This relates to the availability of staff and the degree of burden that financial management systems can cause for individual health workers and the facility staff as a whole (KII 8). In these situations, management tasks can take away staff from essential clinical work. However, others stressed that management is inherent to, and essential for, better clinical performance (KII 3).

Another issue is that of the limited capacity and skills of health staff and in-charges, as well as of facility management committees, in relation to management, accounting and information technology (KII 4, KII 7, KII 8). Likewise, the Lancet Global Health Commission on financing primary health care stresses that fostering provider autonomy requires building capacity and skills for management, in particular where funding flows are fragmented and subject to complicated rules to account for each separately. Where providers do not have the skills to manage new procedures, the results of new purchasing and payment methods will be either diminished or perverse [19]. A study in Zambia found that PBF can improve financial autonomy and accountability, but the change is heterogeneous, indicating that managerial styles, capacity and skills matter, as well as the operating environment [11].

On the other hand, it is also argued that the skills required are relatively basic at primary care level and not dissimilar to managing a household budget (KII 3), and training can be provided to facility managers. For example, Tanzania opted to build capacity with an on-the-job training approach, running in parallel to the launch of the new accounting and reporting systems (KII 3, KII 4). Yet another (or additional) option is to place accountants to work with one or a group of facilities, either directly deployed by central level or locally recruited by facilities. However, these options are often resource and time-intensive and require high level of (external) technical and financial support, which might not be replicable in all contexts, and often capacity challenges remain.

Overall, there is need to carefully consider when additional autonomy makes sense. At very low system level, for facilities with very few staff (for example, dispensaries), the requirements of autonomy and resources needed to make it function (for example, additional training and/or accounting staff) might overweight the benefits that autonomy might entail for the facility (KII 11).

#### Implementation issues and contextual factors

Literature and interviews presented examples of cases where, while autonomy for primary care providers was formally available, in practice conditions or implementation issues strongly limited or impeded its realisation. Experts stressed that the exercise of financial autonomy is highly contextual (KII 2, KII 6). Local legacies, such as administrative structures established during colonialism and persevering long into post-independence, or the legacies of the Soviet system in East European and Central Asian countries, for example, have a deep influence on willingness to exercise facility autonomy, as well as on the culture around autonomy for facilities (KII 3). Another common issue is when providers are grossly underfunded and/or the majority of their budget is allocated to human resources, thus reducing or eliminating the discretion of its use. This is the case, for example, in Cameroon [42], where managers complained about the lack of funds to manage.

Fragmentation in funding makes financial flexibility and autonomy quite complex to realize and there is also a concern about the complexity of financial rules and regulations (either in the PFM systems or introduced by other reforms) and whether they are proportionate for what are often relatively small amounts of funds that are available to manage at primary care level [50]. In some cases, very detailed guidelines are given despite the low amount of funds (and therefore limited risk) and the issues engendered by the complexity are compounded by the staff, time and skills availability (or lack thereof) mentioned above (KII 8). Tight controls and strict guidance is not only an issue in terms of how time-consuming and complex it is for staff, but can also limit flexibility as often there are onerous pre-authorisation processes for reallocation of even small items (KII 7). This limits the funding *de facto* available for facilities and might lead to negative consequences such as rationing, lower quality and responsiveness, and introduction of informal fees.

The empirical literature presents some examples of this situation. In Indonesia, the national health insurance system pays primary care providers by capitation, but there are strict rules about how they can allocate those funds between staff payments and other operational costs. In addition, facilities which were granted autonomy following a PFM reform have four separate bank accounts and must allocate and account for them separately. As a consequence of these challenging rules, funds are rarely used and often sent back to Treasury (KII 5) [40, 50]. Similarly, in Benin, Zambia and Zimbabwe, it was found that PBF funds were often left unspent because of the lack of understanding of providers of the complex rules, as they were not fully aware of their discretion or feared repercussions in case rules were not fully followed [51–53]. Especially where rules are complex, “sense-making” by key actors (how they understand and interpret facility autonomy) defines its de facto implementation [1]. In Kyrgyzstan, providers should receive a capitation payment in instalments and report expenses. In practice, the National Insurance imposes the submission of a monthly report detailing the forecast expenses in order to release the monthly payment. As a consequence, providers’ monthly spending cannot vary much from the historical spending, and most is spent on HRH [54].

Examples of settings where more flexibility has been allowed to address complex and time-consuming rules include the Solomon Islands, where the MoH issues *imprest* (cash advance) accounts at the National Treasury – which enable staff to pay for outreach, supervision and training where procurement in advance can be difficult – and are replenished upon submission of expenditure reports and supporting documents. Lao also uses imprest accounts, but only for donor-funded programmes (KII 7).

### Impact of changes in the scope of financial autonomy for primary care providers

Overall, evidence on the impact of changes in the degree of facility autonomy on providers and service delivery is limited. It is also difficult to disentangle the effects of facility autonomy from those of the other changes introduced by the reforms, such as increased funding, performance objectives, and new management tools and training, alongside contextual factors that affect design and implementation [1]. Experts noted that evidence that positive impacts are linked exclusively to autonomy is weak and alternatives such as funding held at district level and used on behalf of the facility work well in some settings, such as in Sri Lanka and Bangladesh (KII 6). In addition, the literature focuses on a few well-studied country examples (many co-authored by project implementers), while other country experiences are less well evaluated and published (likely due to the absence of substantial external programme support). However, the literature identifies some potential (positive and negative) impacts, which we summarise here.

First, financial autonomy can lead to better availability of drugs and other commodities. In Cameroon, a study noted that the change in availability of essential medicine following the introduction of a PBF scheme resulted from several pathways, including greater autonomy of facilities, enforced regulation from the district medical team, greater accountability of the pharmacy attendant and supply system liberalization which allowed private actors to supply drugs to PBF facilities. However, implementation challenges, such as delays in PBF payments, limited autonomy, lack of leadership, hindered further improvements and led to heterogeneity in performance between health facilities [55]. In Uganda, a study (on autonomy broadly and not exclusively on financial autonomy) found increased decision space was significantly positively associated with some indicators, such as essential drug availability, but not others, such as performance management and quality improvement measures. The conclusion was that increasing managerial autonomy alone is not sufficient for improving health facility performance and that other factors and (pre)requirements mediate relationships between decision space and performance [56].

Financial autonomy could lead to improved motivation of staff, because of their ability to exercise decision power but also because of a better working environment, such as available drugs, staff and incentives, although this is mediated by increased funding. In the case of Tanzania, two studies provide evidence of increases in the motivation of health workers and managers due to the increased financial autonomy of the facility with PBF and DFF schemes [57, 58]. In Zambia, the PBF group had higher satisfaction than both control groups (funding only and business as usual) because they have not only a higher compensation but also financial autonomy, which was part of the PBF intervention [51]. In Kenya, two studies identified similar patterns [59, 60], while another noted that lack of autonomy leads to demotivation among staff, for example when collected resources such as user fees are transferred to local governments [61].

Financial autonomy may stimulate health facility governing committees that were otherwise inactive, and therefore support community participation and (coupled with flexible use of resources) also providers’ responsiveness. This is the case of Burundi with the introduction of a PBF scheme, which entailed increased autonomy [62]. Similarly, in Tanzania, the functionality of Health Facility Governing Committees improved with DFF [63]. Oversight mechanisms can be designed to allow for increased social accountability and community participation, for instance by enhancing the role of Health Facility Committees [64]. In Tanzania, it was found that with DFF and increased autonomy over financial management, the functioning of Health Facility Governing Committees improved and they displayed better levels of accountability to the community on issues such as availability of medicines and commodities, and provision of timely care [63, 65]. Vice versa, findings from Kenya highlighted how Health Facility Management Committees can strengthen autonomy in practice, when they are equipped with clearly defined roles in financial supervision and governance [60]. However, another expert cautioned that increases in facility autonomy do not guarantee the effectiveness of community structures for participation, as such systems for downward accountability might be legally in place but not effective or appropriate (KII 8). In addition, complex accountability structures may burden facilities.

There is some limited evidence of increase of access to health care due to reforms which included increased financial autonomy (among other elements and notably increased funding). In Tanzania, for example, deliveries occurring at health facilities increased by 33.6% (*p* < 0.001) one year after the DFF implementation [66]. In Argentina, the ‘Plan Nacer’ increased the use and quality of prenatal care [67]. This increase in the quantity and quality of outputs was explained by, among other factors, higher flexibility in the use of resources at the facility level [68].

In line with this, other studies highlight the increase in efficiency of service delivery which could be spurred by improved financial autonomy. In Kenya, different stakeholders in the health system identified financial autonomy as one of the relevant factors to improve efficiency, which provided increased agency to managers to address emergent issues [69]. The lack of financial autonomy (understood as centralisation of resources) was identified as a driver of inefficiencies in Kenya. This flows from lack of predictability of the access to resources, corruption in procurement processes, the bureaucratic and high burden of accessing resources, and the return to an input-based budgeting, which breaks the direct link between desired results and budgets [69, 70].

Although the literature is mostly focused on reporting success stories, some examples of negative implications of financial autonomy for providers and service delivery are discussed. Some are linked to design and implementation issues which we presented above, such as delays in payments, causing staff frustration and demotivation [38, 71]; increased workload for facility staff due to complex accounting and reporting requirements [72]; and other complex rules that lead to unspent funds (54,80,81).

There is also some evidence of perverse incentives or extractive practises, such as rent seeking, cost escalation, selling of branded medicines, and in general profit maximising or extractive strategies (when relying on user fees), but these are typically focused on the hospital sector. However, they constitute a risk also at primary care, especially in the context of resource constraints (KII 6, KII 8) [17].

A key lesson from the COVID-19 crisis is the importance of health facilities enjoying adequate autonomy to respond to health emergencies. Such autonomy empowers healthcare service providers to respond more swiftly and flexibly to emerging health needs. An example can be found in Indonesia, where during the COVID-19 pandemic, BLUD facilities (enjoying a larger degree of financial autonomy) were able to utilise the national health insurance (*Jaminan Kesehatan Nasional*) capitation funds for both routine essential health services and emergency service provision and performed better (KII 10). In contrast, regular non-autonomous public providers faced challenges in promptly accessing increased public funds, thereby hindering their capacity to provide essential services during COVID-19 [73].

## Discussion

### Typology of levels and features of financial autonomy

Based on the different aspects of financial autonomy for primary care providers in LMICs identified from our literature review and expert consultation and the need to distinguish them (and fit them to context), we developed a simple typology, structured by the different stages of the budget cycle (Table 1). This is a useful starting point for a country’s assessment of its financial autonomy aspects. In fact, the most common scenario in LMICs is the middle one, though countries (and facilities within them) can combine different features across the budget cycle stages. We recognise that facilities typically have multiple, fragmented financial flows from different purchasers, and as such, rules on management (including planning, expenditure, reporting, etc.) may differ and as a consequence there can be different levels of autonomy for the same facility, based on the funding source.

**Table 1:** Typology for financial autonomy at primary facility level

	Low financial autonomy scenario	Medium financial autonomy scenario	High financial autonomy scenario
Planning of budget	Budgets are allocated from above with no scope for facilities to influence them	Facilities make inputs into budget process but can only influence the final budget in limited ways	Facilities structure own budgets according to their identified activities and needs
Mobilising and retention of additional funds	Funds are fixed externally; no ability to mobilise additional funds at facility level. Funds remitted to Treasury or district/higher level. All funds spent within financial year	Most funds are fixed; some small (marginal) additional fund mobilisation is permitted and retained at facility level, with rest remitted to higher levels. One part of revenues can be retained (e.g. use of user fee or PBF income) across years	Able to raise funds independently from multiple sources, as available, without restrictions. All funds raised are retained at facility level. All funds can be retained across years, if unspent
Management, including reallocation	Budgets are fixed (often by line item) and changes across them are very cumbersome and limited (or not possible). Most of expenditure is ring-fenced. Where multiple revenue sources exist, there are strict rules about how they can be used	Some in-year changes in budget are possible, with higher authorisation. There is some flexibility around deployment of different revenue streams according to facility needs	Facilities can shift funds across budget lines within clear parameters set out in advance (with simple approval procedures where this threshold is exceeded), drawing flexibly from the different funding streams that they can access
Expenditure	Most expenditure is made at higher levels (on behalf of the facilities), with inputs provided in kind. Facilities do not need or have bank accounts	Facilities have access to limited funds to use for small costs (often minor operational costs, such as cleaning and maintenance). They may have bank accounts but can also operate through earmarked funds held at the district, petty cash or mobile funds.	Facilities can actively manage their major expenditure items, including for locally hired staffing, medicines and supplies and operational costs. They all have bank accounts
Reporting	Facilities have no financial reporting requirements as they are not recognised within the FMIS system	Facilities report on expenditure through a simpler tool, which is integrated into the FMIS via higher levels (such as districts) for funds released by them to the facilities	Facilities are spending units, accounting within the FMIS for their expenditure

*Source: authors*

It is also interesting to note that having more payers and different rules mean that providers can move funds around and substitute funds, i.e., use more regulated funds for certain expenses, and keep less regulated ones for others, in order to optimise the allocation of resources while following the rules. Usually, the most flexible funding sources are those received locally and directly, such as payments from insurance or other coverage schemes, or often user fees. For such locally generated revenues, there is often a very high level of autonomy in spending as there are weaker reporting or monitoring requirements, unless the facilities are mandated to remit these revenues to Treasury or to the district or county. These can represent a high proportion of fungible revenues at facility level in many contexts. In Ethiopia, for example, primary care facilities are permitted to retain revenue generated from user fees, whereas resources obtained from alternative channels are subject to stringent reporting obligations and, if unutilised, refunded to the purchaser [74, 75]. Payments from non-governmental organisations or donors, including PBF, allow autonomy but usually within limits (with proportions earmarked for specific purposes, such as staff bonuses), while government budget payments are often fully earmarked (for salaries, drugs, infrastructure, etc.).

### Conceptual framework

**Fig. 2:**
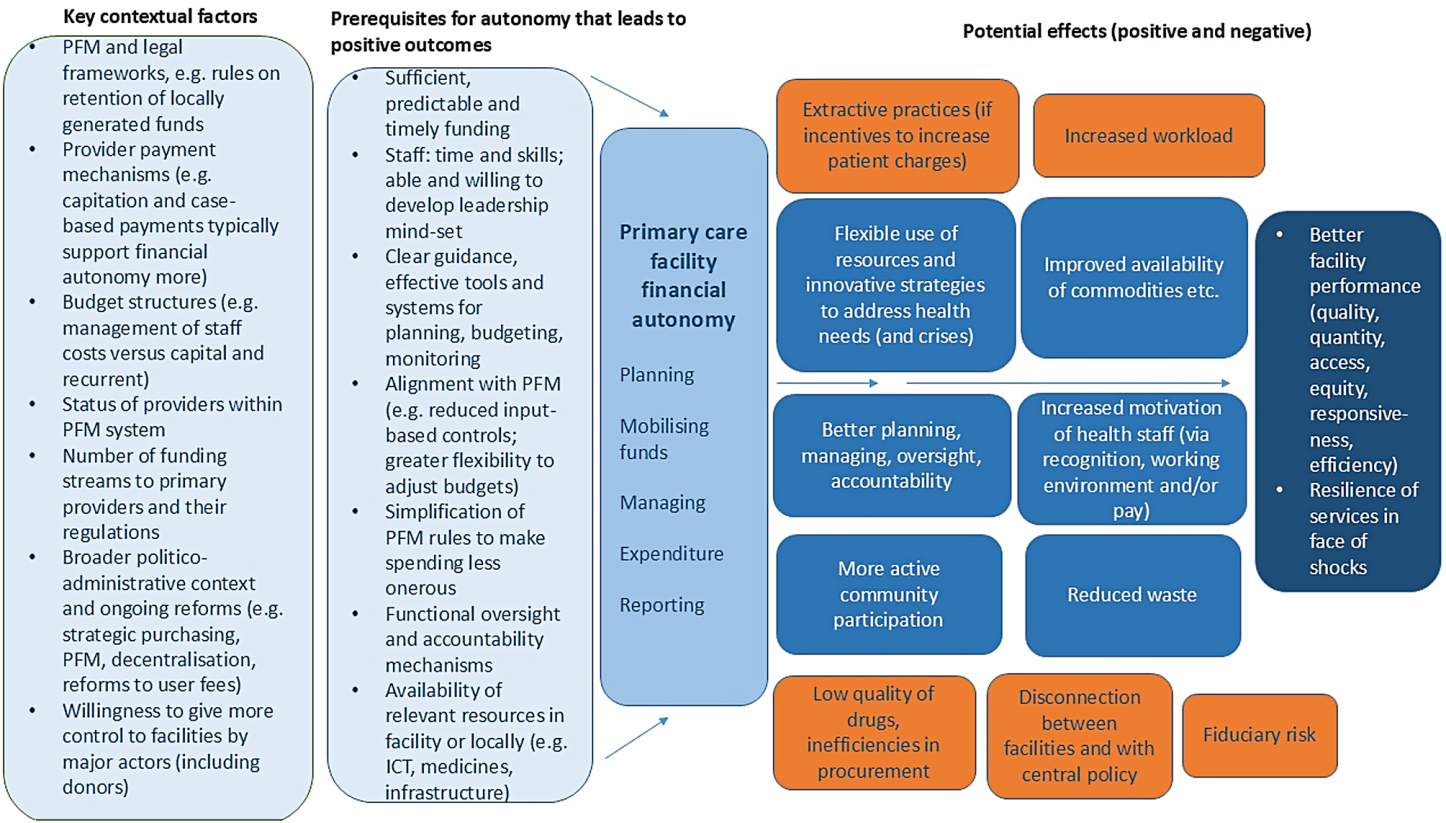
Conceptual framework for primary care facility financial autonomy. Source: authors

Synthesising the above findings, we present a conceptual framework highlighting some key factors emerging that influence and are influenced by financial autonomy for public primary providers (Fig. 2). These include contextual factors, such as the status and level of the provider, administrative context (such as decentralisation) and provider payment arrangements.

In relation to the contextual factors, the status of primary care providers varies vastly along a spectrum, from being entities of the MoH (with no independent budget status) to being completely separate budget units, with a range in the middle consisting of a certain degree of autonomy (defined by laws and regulations) and recognition in the PFM system. Different ways of purchasing health services and paying providers also affect and are affected by the autonomy of primary care facilities. For example, strict line-item budgets do not allow financial autonomy for primary care facilities. In contrast, mechanisms such as capitation or fees for service (as used in many PBF projects, with some adjustments) typically permit more financial autonomy and flexibility of providers to use the funds they receive. However, rules and guidance or implementation issues might constrain the actual autonomy of providers.

The framework also highlights preconditions or prerequisites for success. One is that autonomy is accompanied by sufficient funding to primary care facilities to avoid extractive practices noted at hospital level, and (ideally) funding that can be pooled at facility level and used following one set of PFM rules. Other prerequisites such as management time and skills, clear guidance and tools for fund use, alignment with PFM regulations, functional accountability mechanisms and availability of relevant inputs such as drug supplies and IT systems are also highlighted.

A core underlying hypothesis is that primary care facilities are best placed to know the combination of resources and inputs that they need in order to provide services that address the needs of their community, both in terms of treatment, but also in relation to the active management of community health (preventative and communication/education activities). On the right-hand side, the framework outlines the potential effects, both positive (dark blue boxes) and negative (orange boxes), as well as risks. Financial autonomy can contribute to better facility performance in terms of quantity of services produced (incentivising use of services by community members) and quality of services provided. Mechanisms here can include reduction of stock-outs and improved availability of commodities, including (in some cases) medicines which facilities might be able to procure directly. Another potential mechanism is through increased motivation of staff because of the improved working environment and, potentially, greater recognition of their professional autonomy, and (in some cases) payment of staff bonuses,

Through reinforcement of the role of health facility committees to manage funds, and strengthened community participation, responsiveness of service delivery may also be improved. In addition, because primary care facilities decide autonomously how to use resources, rather than being provided inputs that they might not require, autonomy can lead to more efficiency, less waste and extra funds, which can be reinvested in the facility. Creation of new PFM systems and provision of tools for planning, budgeting and management (as well as reporting), alongside sufficient training, technical infrastructure and capacity to use them, allows further improvement in the efficiency of service delivery. Despite reporting and oversight mechanisms being in place, however, increased facility autonomy does entail the possibility of misallocation of funds and increased fiduciary risks – although this is limited as funds at primary care level are often relatively low. Autonomy in drug procurement might also entail issues with quality and inefficiencies. Similarly, autonomy might mean an increased workload for staff, leading to their demotivation (as outlined above).

If all (pre)conditions are in place and linkages fully functional, the improved quality, quantity, perhaps availability (if facilities have incentives to improve their range of services), efficiency and responsiveness would contribute to better population health outcomes. However, as described above, the evidence for the strength of these links is varied and broadly limited, and it is clear that facility autonomy is not sufficient on its own to lead to such outcome changes.

### Study limitations

Some important limitations of the study should be noted, including the scoping nature of the review, and the challenge of disentangling effects from the other components that typically accompany financial autonomy. We also note that we focused on the public sector. Private and faith-based facilities generally have greater financial autonomy compared to public facilities, as they are not bound by PFM rules. The focus on the public sector may have left out important findings and lessons.

### Areas for future research

This review highlights a gap in the literature, in that no dedicated studies on provider autonomy reforms at primary care level in LMICs were identified (with financial autonomy only briefly discussed in studies on wider reforms, such as purchasing). Many studies refer to financial autonomy but few go into any detail on it. One challenge is that for a literature search, there is no Medical Subject Headings (MeSH) term for provider autonomy (much less financial autonomy); the available MeSH term of ‘professional autonomy’ relates to clinical aspects. This makes evidence synthesis in this area challenging. There is need for more in-depth case studies to be undertaken to provide richer evidence from a wider range of contexts on the nature and strength of the relationships in the proposed conceptual framework that has been elaborated here. The proposed conceptual framework as well as the typology provide orientation for such studies.

Autonomy is not a goal in its own right, but a means to an end (of better performance of primary care facilities and better health outcomes, including preventative), so reforms should be monitored to track these important outcomes. Other more specific areas to explore include different ways of increasing primary care financial autonomy without high transaction costs; better documenting the role of multiple funding flows at primary care level, and the level of autonomy providers have over these; and understanding what degree of autonomy best fits different expenditure categories (e.g. management of staffing budgets) and facility. Further work could include deepening the conceptualisation of different aspects of financial autonomy (identifying which are most crucial), along with more empirical studies of their effects.

It is interesting to compare the findings on financial autonomy level with those from reviews focused on autonomy in the hospital sector in LMICs [17], where different motivations – including seeking to relieve pressure on public budgets – emerge as significant. The mechanisms of change also diverge, with privatisation and corporatisation being some of the common reforms which increase hospital financial autonomy. Mixed results are highlighted by a recent review of hospital autonomy [17], which in common with ours highlights the importance of management strengthening as a precondition for success, along with good preparation and the engagement of all stakeholders prior to changes. As noted in the limitations, more lessons for the public sector could be derived from reviewing the literature on the financial autonomy of the private sector.

## Conclusion

This article explores a topic which has been rising up the agenda in terms of global health discourse, and yet which is rarely unpicked in detail, especially in relation to primary care. It has mainly been addressed in literature on strategic purchasing and on PFM, but typically these approach from different perspectives (one more positive and the other more cautious) and have not been explored together.

We bring together some of the key contextual factors that influence the degree of financial autonomy permitted and enacted (including political economy factors), the many prerequisites for it to be fully exercised (including the importance of an active management mind-set and skills, which are often assumed but may not be present or easy to nurture in all environments), some reflections on design, and a number of the main positive effects and risks that have been noted. Our proposed typology can help map more specifically the different dimensions of financial autonomy across the budget cycle. It has policy relevance as a tool for countries to identify whether they are at the right point on the spectrum for their contextual needs and capacities, and plan accordingly. Facilities may have considerable autonomy in one aspect but not another, and their interaction is important. Equally, autonomy often varies according to funding sources and expenditure types, which can be a complex mix at facility level. It is not a binary choice (autonomous or not), nor is it a simple continuum (with more autonomy always being better). The arrangements need to follow the contextual needs.

Reflecting on the evidence and our conceptual framework, it is clear that there are more potential benefits from financial autonomy at primary facility level (if appropriately supported) than there are risks, so there should be a presumption in favour of supporting an increase in this area in most settings where it is currently limited, supporting facilities to deliver more effectively and efficiently within their current mandated service area. Some elements appear to be particularly important to support autonomy, including: 1) the ability to retain at least some funds generated; 2) the ability to shape the budgets that apply to their level; 3) the ability to vire across budget lines within limits that are clear, reasonable and stated in advance; 4) being able to address (at minimum) routine operational costs without prohibitive approvals and accounting. Considering our typology, primary care facilities should in principle find themselves in the middle or high scenarios; the low autonomy one does not offer scope for effective local management of services.

While financial autonomy is *prima facie* a positive attribute - and especially in these crisis-prone times when adaptability and resilience are needed more than ever - the understanding of autonomy *over what* (for example, different funding streams and budget lines), for *which purposes* (for example, at different points of the budget cycle) and *by whom* (the optimal balance of responsibilities across levels, taking into account contextual factors, including facility size) is still not clearly addressed in the literature, along with the implications for PFM and other system components (including community health systems) and the wider benefits and cost of reforms. These should be the focus for future research, which can be informed by and further develop our conceptual framework. We highlight the need for better conceptualisation: autonomy has many aspects, which need more detailed unpicking, potentially along the lines of the typology which we have developed here.

## Electronic supplementary material

Below is the link to the electronic supplementary material.

Supplementary material 1

## Data Availability

The datasets used and/or analysed during the current study are available from the corresponding author on reasonable request.

## Footnotes

1 Financial autonomy of public primary care providers: What are the issues?

## Abbreviations

DFF: Direct facility financing
FMIS: Financial management information system
HRH: Human resources for health
KI: Key informant
LMIC: Low- and middle-income countries
MoH: Ministry of Health
PBF: Performance-based financing
PFM: Public financial management
WHO: World Health Organization
