# Supplementary material for: Financial autonomy of facilities providing primary care services in low- and middle-income countries: assessing the evidence to inform the development of a typology and conceptual framework
Source: BMC Health Serv Res. 2025 Dec 15;26:92. doi: 10.1186/s12913-025-13863-7 (PMC12822310; doi:10.1186/s12913-025-13863-7)
Supplement: Supplementary file 1 — Supplementary material 1 [file 12913_2025_13863_MOESM1_ESM.docx]

Expert consultation: financial autonomy at primary care level

## Guide

*Our team is working on a literature review about financial autonomy of primary care health providers in low- and middle-income countries for the World Health Organisation. We aim to gather and synthesise evidence on the topic to inform future research and policy.*

*We are making a round of consultations to experts, either from academia, donors, and policy makers in areas such as health systems, health financing and policy reforms.*

*We would like to ask you some questions so you can provide further information and opinions on the matter.*

1. Name, position, and institution

**Context**

1. Which countries/regions and timeframe does your experience in relation to financial autonomy and related reforms refer to?
2. [in each country or broadly if you see any patterns across countries] what was the broader background to the reform? Why was autonomy changed for facilities providing PHC? What were the drivers of the reform? (for example, PBF/DFF, PFM changes, accountability and community participation, etc.)

**Requirements / design of reforms**

1. [with reference to one country in turn] Could you briefly explain in what the changes consisted? Was autonomy increased or decreased? What were the **key strategies** or **mechanisms** that were introduced to modify the level of financial autonomy for PHC facilities?
   1. Examples include: higher level changes for example to budget cycle, budget flows, PFM systems, or implementation mechanisms, modifications to payment methods, purchasing arrangements, laws and regulations, contracting and management actions, etc.
2. How did the implementation of reforms that affect financial autonomy go? Have there been any specific **challenges or barriers** encountered during the implementation of financial autonomy, and how have these challenges been addressed?
   1. In particular, the literature (e.g. on PBF) notes some potential barriers to autonomy – do these resonate/could you elaborate on those in the context? [or can be used to probe further after question above:
      1. Discrepancy between autonomy on paper and de facto/the reality, due to complex rules and regulations
      2. Lack of managerial and financial skills for health staff/in charges and/or facility management committees has also been identified as a barrier
      3. Lack of actual resources to manage
3. in contrast, what are the facilitating factors – for example, **key requirements or prerequisites** that were introduced or were already existing and contributed to increased financial autonomy in primary care health facilities?
   1. What are the lessons learned in terms of changes or adjustments need to be made in terms of governance, financing, and management structures?
4. In your experience, which countries/contexts were more successful in making effective changes to financial autonomies for PHC facilities, and why? what contributed to success?
5. Has COVID-19 (and the challenges and adjustments it has demanded) had an impact as driver of reforms and changes to financial autonomy? If so, how?

**Effects / implications**

1. What are the **implications** of changes to the level of financial autonomy (both positive and negative)? What specific changes or transformations have occurred as a **result** of financial autonomy in primary care health facilities *(Probe about efficiency, effectiveness, and sustainability of the facilities – also could be negative)*
   - 1. provider performance,
     2. efficiency in spending,
     3. clarification/changes in roles and responsibilities of implementing entities,
     4. strategic planning,
     5. prioritization,
   1. [if they are referring to ToC only, probe] Do you have specific examples at country level?
2. What have been the **outcomes** and **impacts** of the financial autonomy reforms
   1. On service delivery: efficiency, quality of care, equity in access of services provided by primary care health facilities? (*Probe - also could be negative)?*
   2. In terms of resource allocation, decision-making, transparency and accountability, and fiduciary risk? *(Probe about access to funds, management, allocation, and expenditure funds)*
   3. Have there been any notable innovations or best practices that emerged as a result of these changes?
3. What are the **implications for the wider health system**? How does the level of financial autonomy influences or is influenced by factors such as health financing mechanisms, payment systems, governance structures, information systems, and human resources for health?

**Recommendations**

1. Can you recommend to us academic or grey **literature** on the topic in addition to our list which we can share?
2. Can you recommend other experts at country level or globally on this topic?
